# Supplementary material for: Previous Usutu Virus Exposure Partially Protects Magpies (Pica pica) against West Nile Virus Disease But Does Not Prevent Horizontal Transmission
Source: Viruses. 2021 Jul 20;13(7):1409. doi: 10.3390/v13071409 (PMC8310384; doi:10.3390/v13071409)
Supplement: Supplementary file 1 [file viruses-13-01409-s001.zip › viruses-1270571-supplementary.pdf]

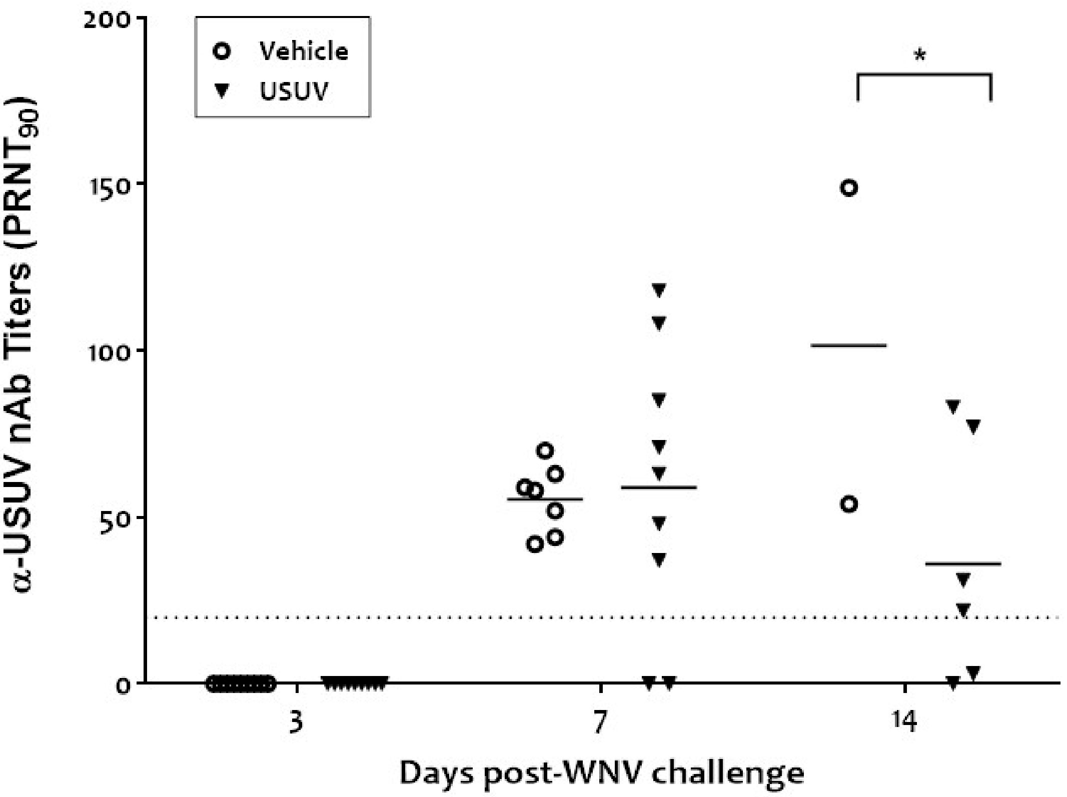

Figure S1. Neutralizing antibodies against USUV developed by experimentally WNV-infected magpies.
